# Time and Frequency-Dependent Modulation of Local Field Potential Synchronization by Deep Brain Stimulation

**DOI:** 10.1371/journal.pone.0102576

**Published:** 2014-07-16

**Authors:** Clinton B. McCracken, Zelma H. T. Kiss

**Affiliations:** Department of Clinical Neurosciences and Hotchkiss Brain Institute, University of Calgary, Calgary, Alberta, Canada; Federal University of Rio Grande do Norte, Brazil

## Abstract

High-frequency electrical stimulation of specific brain structures, known as deep brain stimulation (DBS), is an effective treatment for movement disorders, but mechanisms of action remain unclear. We examined the time-dependent effects of DBS applied to the entopeduncular nucleus (EP), the rat homolog of the internal globus pallidus, a target used for treatment of both dystonia and Parkinson’s disease (PD). We performed simultaneous multi-site local field potential (LFP) recordings in urethane-anesthetized rats to assess the effects of high-frequency (HF, 130 Hz; clinically effective), low-frequency (LF, 15 Hz; ineffective) and sham DBS delivered to EP. LFP activity was recorded from dorsal striatum (STR), ventroanterior thalamus (VA), primary motor cortex (M1), and the stimulation site in EP. Spontaneous and acute stimulation-induced LFP oscillation power and functional connectivity were assessed at baseline, and after 30, 60, and 90 minutes of stimulation. HF EP DBS produced widespread alterations in spontaneous and stimulus-induced LFP oscillations, with some effects similar across regions and others occurring in a region- and frequency band-specific manner. Many of these changes evolved over time. HF EP DBS produced an initial transient reduction in power in the low beta band in M1 and STR; however, phase synchronization between these regions in the low beta band was markedly suppressed at all time points. DBS also enhanced low gamma synchronization throughout the circuit. With sustained stimulation, there were significant reductions in low beta synchronization between M1-VA and STR-VA, and increases in power within regions in the faster frequency bands. HF DBS also suppressed the ability of acute EP stimulation to induce beta oscillations in all regions along the circuit. This dynamic pattern of synchronizing and desynchronizing effects of EP DBS suggests a complex modulation of activity along cortico-BG-thalamic circuits underlying the therapeutic effects of GPi DBS for conditions such as PD and dystonia.

## Introduction

High-frequency electrical stimulation of specific brain regions, known as deep brain stimulation (DBS), is an effective treatment strategy for a number of refractory neurological conditions. DBS of the globus pallidus internus (GPi) provides significant symptom relief for both Parkinson’s disease (PD) and dystonia [1], [2], however, despite widespread clinical use, consensus regarding the therapeutic mechanisms of action is lacking.

Initial clinical reports that the effects of DBS appeared to be qualitatively similar to those produced by a lesion of the same region [3], [4] led to the idea that DBS exerted its effects by creating a reversible “functional lesion” of the stimulated nucleus. A variety of animal studies demonstrated that DBS inhibited activity in the stimulated region, through either depolarization blockade, neurotransmitter depletion, or enhanced local GABAergic transmission [5]–[9]. However, recent evidence suggests that other mechanisms may also be important. Electrical brain stimulation at clinically effective intensities preferentially excites axons as opposed to cell bodies [10], such that activation of afferent and efferent axons can modulate neuronal activity in sites distal to the stimulated nucleus [11]–[15]. Thus, GPi DBS can both inhibit local firing [5], [16], [17] as well as activate efferent GPi axons projecting to thalamus [18]–[20].

Both dystonia and PD show evidence of pathological hypersynchrony in local field potential (LFP) oscillations in the basal ganglia (BG) and cortex (Brown et al. 2001; Chen et al. 2006b; Weinberger et al. 2012), and reduction of these oscillations has been suggested as a potential mechanism of DBS [21]. How DBS affects LFP oscillations in intact animals with no obvious pathology is not known, and assessing the effects of DBS in normal animals is critical for informing the interpretation of the effects produced in disease states (Chiken and Nambu, 2003). This is particularly true when considering that PD and dystonia, both effectively treated by GPi DBS, are hypo- and hyper-kinetic movement disorders, respectively, with different pathophysiologic features [22], [23]. Mechanistic studies have typically examined the effects of GPi DBS acutely over seconds [9], [24]–[26]; however, how the immediate effects of stimulation relate to clinical improvement is not clear. Although the therapeutic effects of GPi DBS on PD symptoms occur very rapidly [27], maximum clinical benefit for dystonia can take much longer, i.e., weeks to years [28]–[32]. Accordingly, clarifying how the effects of DBS evolve over time is a high priority. Furthermore, many studies have not controlled for non-specific effects of stimulation by comparing the effects of low-frequency (LF) to therapeutic, high frequency (HF) stimulation. LF DBS generally does not produce beneficial effects and in some cases may be deleterious [33]–[38]; it is therefore important to identify changes in neural activity that are specific to high-frequency (i.e., therapeutic) stimulation.

Given the evidence for circuit-wide effects of DBS, we examined how DBS delivered to the entopeduncular nucleus (EP; the rat homolog of the primate GPi) affected spontaneous and evoked synchronous LFP activity both within and between a number of regions comprising the primary motor circuit. We recorded LFP activity simultaneously from the dorsal striatum (STR), primary motor cortex, (M1), and ventroanterior thalamus (VA), while DBS was applied to EP for 90 minutes, with short breaks after 30 and 60 minutes to assess activity induced by acute EP stimulation. Given the hypersynchrony associated with PD and dystonia, and the beneficial effects produced by GPi DBS, our working hypothesis was that the EP DBS would be globally desynchronizing. Although we saw widespread reduction in low beta synchronization both within and between regions, we identified more complex and time-dependent changes in both power within and functional connectivity between regions.

## Methods

All procedures were performed in accordance with Canadian Council for Animal Care guidelines, and were approved by the Institutional Animal Care and Use Committee of the University of Calgary.

### Animals and surgery

Male Sprague-Dawley rats (275–400 g) were anesthetized with urethane (1.5 g/kg, i.p.) and placed in a stereotaxic frame. Body temperature was maintained at 37°C with a temperature-controlled heating pad. In all surgical preparations the scalp was exposed and burr holes were drilled in the skull overlying M1, dorsocentral striatum, VA, and over the contralateral cerebellum to allow for insertion of the stimulating electrode into EP at a 30 degree angle to avoid potential damage to EP-VA connections. A concentric bipolar stimulating electrodes (SNEX-100; Kopf, Tujunga, CA) was placed in EP - anteroposterior (AP) −3.0 mm (from bregma), mediolateral (ML) +2.6 mm, dorsoventral (DV) −8.0 mm (vertical from skull); epoxy-insulated tungsten recording electrodes (0.125 mm shank diameter, 0.8–2 MΩ impedance at 1000 Hz, FHC, Bowdoin, ME) were slowly lowered into M1 (AP: +2.2 mm, ML: +2.8 mm, DV: −2.2 mm), striatum (AP: +1.2 mm, ML: +2.8 mm, DV: −5.0 mm) and VA (AP: −1.8 mm, ML: +1.8 mm, DV: −6.0 mm). Following implantation, electrodes were allowed to settle for at least 20 minutes before recording commenced.

### Recording

**Figure 1:**
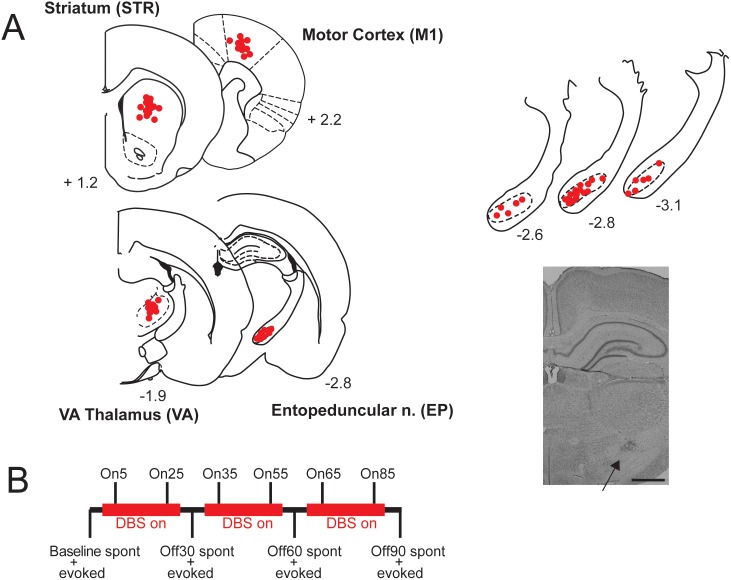
Electrode placements and experimental design. **A:** Left: Dots show recording electrode placements in striatum (STR), primary motor cortex (M1), and ventroanterior thalamus (VA) and DBS electrode placements in entopeduncular nucleus (EP), and; numbers represent antero-posterior distance from bregma. Overlapping placements have been omitted for clarity, and placements have been collapsed into the same plane and may be slightly anterior or posterior (±∼200 µm) to the indicated distance from bregma. Right: Enlarged sections showing placements in EP and photomicrograph showing marked electrode location in EP. Scale bar = 1 mm. **B:** Outline of stimulation and recording protocol.

LFP signals from the recording electrodes were amplified (gain: 1000), analog filtered (0.1–1000 Hz) by a multichannel amplifier (A-M Systems) and displayed on an oscilloscope (Tektronics, Wilsonville, OR). The data were digitized at 10 kHz using an Axon Digidata 1440 (Molecular Devices, Sunnyvale, CA), acquired using Clampex 10.2 (Molecular Devices), and stored for off-line analysis. LFP signals were referenced to a skull screw over the contralateral cerebellum. DBS was applied to separate groups of rats at 130 Hz (HF) or 15 Hz (LF) using parameters (80 µA, 0.2 ms pulse duration) that preliminary experiments indicated was below the threshold for evoking a motor response. Given the electrode surface area, the selected current intensity generates a charge density (20 µC/cm^2^/phase) that approximates that which is clinically effective in humans [39], and shows effects in various rat behavioral studies [40], [41] while remaining below the recommended clinical safety limit of 30 µC/cm^2^/phase [42]. To control for the effects of electrode implantation and surgery, another group had the DBS implanted but did not receive stimulation (SHAM). DBS was applied for three 30 minute sessions for a total of 90 minutes; sessions were separated by 6 minute intervals where spontaneous and evoked LFP data were sampled with DBS off. Figure 1 shows electrode placements, and a schematic of the recording procedure.

We analyzed both spontaneous LFP oscillations and those induced by single-pulse stimulation of EP to address separate dimensions of neural activity. Spontaneous recordings assess the state of the system at rest, whereas evoked/induced activity provides an index of how the system responds to activation (analogous to an organism reacting to a stimulus). For spontaneous recordings, LFPs were recorded for 3 minutes at a number of different time points with DBS on or off (Fig. 1). We recorded baseline (BL) LFPs, LFPs with DBS ON during the first 5 and 25 minutes of DBS (referred to as On5 and On25), and then with stimulation OFF after 30 minutes of DBS had been applied (Off30). This protocol was repeated twice to examine changes over longer time periods, giving the additional time points of On35, On55, Off60, On85 and Off90. During OFF periods, spontaneous LFP data were recorded, followed immediately by evoked data, after which DBS was turned ON again. For induced LFP responses, EP stimulation was delivered at 0.4 Hz (600 µA, 0.2 ms pulse duration, 20 stimulation sweeps). This stimulus intensity was chosen based on preliminary experiments, with this intensity being the lowest that consistently resulted in an oscillatory response. LFP data were recorded for 1 s pre- and post-stimulus. Induced activity was reassessed following 30, 60 and 90 minutes of DBS.

### Histology

At the end of each experiment, small lesions were made at the tip of the electrodes (250 µA, 10 sec current pulse) and visualized by adding potassium ferrocyanide during post-fixation. Animals were euthanized with an overdose of urethane and decapitated. The brain was removed and fixed for at least 48 h in 8% w/v paraformaldehyde (in PBS) and cryoprotected in 25% w/v sucrose (in PBS). Brains were then sectioned (50 µm coronal sections), placed on gelatin-chromalum-coated slides, and stained with cresyl violet for histochemical verification of the recording/stimulation electrode placements (Fig. 1C). Only animals with the cathode located in EP were included for analysis. This pole was also used to record LFP activity from EP when DBS was off.

### Analysis

The spectral power of LFP oscillations in each region was analyzed using routines from the Chronux software package (www.chronux.org) for Matlab (MathWorks, Natick, MA) as previously described [43]. As certain measures of functional connectivity (e.g., coherence) are impacted by volume conduction, we used the debiased weighted phase lag index (WPLI) which is both insensitive to volume conduction effects and more sensitive to true phase synchronization [44] using the FieldTrip software toolbox for Matlab [45]. Before analysis of power and WPLI, signals were processed to remove stimulus artifacts using an offline algorithm (Fig. S1). The algorithm detects stimulus artifacts by thresholding the first derivative of the signal and deleting a defined period surrounding each artifact. The missing values in the signal are then reconstructed using cubic spline interpolation. To ensure this procedure did not introduce any spurious effects or artifacts due to processing, simulated artifacts were added to non-stimulated LFPs (OFF periods and SHAM animals) and then removed using the same algorithm. Furthermore, spontaneous LFP recordings were downsampled to 500 Hz, and segmented (10 s window). Each segment was detrended to remove any slow DC components and padded with zeros to increase frequency resolution.

Multitaper spectral power and WPLI were calculated for each segment in the following frequency bands: slow/delta (0.5–4 Hz); theta (4–12 Hz); low beta (12–20 Hz), high beta (20–30 Hz), low gamma (30–59 Hz) and high gamma (61–90 Hz). The traditional beta and gamma bands were subdivided as reports have suggested differences in generation and function in the low and high beta bands [46]–[48], as well as low and high gamma bands [49]–[52]. Data for each frequency band were then averaged over segments. To compare across different groups and over time, power and WPLI values for each animal in a group were normalized to the mean baseline values for that group. For induced LFP oscillations, power values from the 1 s post-stimulus were normalized to pre-stimulus baseline (1 s) and averaged across stimulation sweeps for each time point. To compare across different groups, the values for each animal in a group were normalized to the mean baseline values for that group.

### Statistics

Changes in spontaneous and evoked power in each region and WPLI between regions due to time and DBS frequency were analyzed using 3-way ANOVA with “stimulation frequency” as a between subjects factor; time and “frequency band” were within subject measures, followed by Bonferroni’s post-hoc test, corrected for multiple comparisons. If the assumption of sphericity was violated according to Mauchley’s test, the Greenhouse-Geisser correction was applied and corrected F values are reported. Significance was set at p<0.05.

## Results

While multisite LFP data were recorded from a total of 36 animals (n = 12/group), all animals with incorrectly placed EP stimulating electrodes were excluded. In the remaining animals, exclusions were made due to incorrectly placed recording electrodes on a region-by-region basis, leaving final group sizes of 6–8 animals/group. In the LF group, DBS stimulation produced acute voltage deflections lasting tens to hundreds of milliseconds (see Figure S1) that corrupted the analysis of oscillations even after removal of the stimulus artifact itself. This did not occur in the HF group, likely due to the short latency between pulses. Accordingly, analysis of the LF group was restricted to acute-stimulus evoked responses in the OFF periods.

### Spontaneous Oscillatory Activity

#### Power

**Figure 2:**
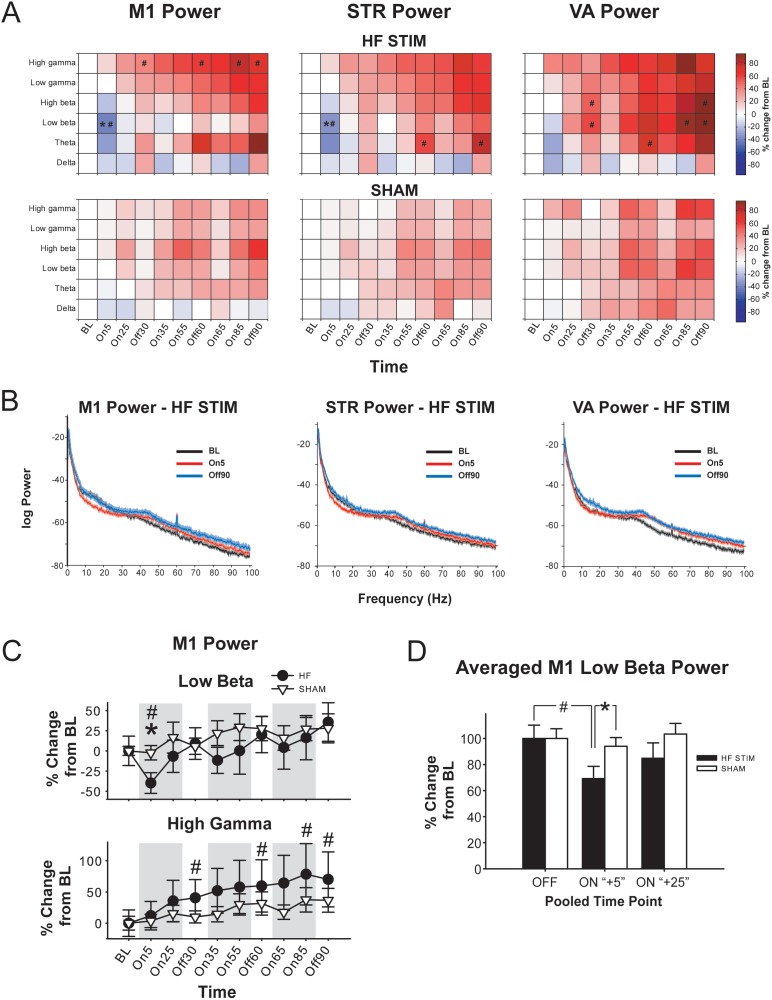
High-frequency (HF) EP DBS produces frequency band-specific and time-dependent increases and decreases in LFP oscillation power in primary motor cortex (M1), striatum (STR) and ventroanterior thalamus (VA). **A.** Color plots showing effects of high-frequency (HF; top) or SHAM (bottom) EP DBS on normalized LFP power in M1, STR, and VA according to frequency band and time point. * = significantly different from SHAM; # significantly different from within-group BL (p<0.05). * = significantly different from SHAM; # significantly different from within-group BL (p<0.05). **B.** Representative power spectra from a rat receiving HF EP DBS at BL, in the first five minutes of stimulation (On5), and after 90 minutes of stimulation (Off90). Shaded area represents 95% confidence interval generated using “leave one out” jackknife statistics. **C.** Effects of HF and SHAM EP DBS on normalized M1 low beta (top) and high gamma (bottom) power over time. Error bars represent S.E.M. **D.** Effects of EP DBS on M1 low beta power, pooled according to relative stimulation time point. OFF = pooled average of BL, Off30, Off60; “ON+5” = pooled average of On5, On35, On 65; “ON+25” = average of On25, On55, On85. * = significantly different from SHAM; # = significantly different from within-group BL (p<0.05).

Analysis of the effects of EP DBS on LFP power in M1 (Fig. 2) revealed that HF DBS significantly reduced low beta power in the first five minutes of stimulation (On5) compared to SHAM DBS at that time point, as well as compared to within-subject baseline (BL) [significant main effect of time (F_3.57, 42.84_ = 12.35, p<0.001) and frequency band (F_2.06, 24.68_ = 6.70, p = 0.004) and a significant time × stimulation frequency interaction (F_3.57, 42.84_ = 2.98, p = 0.036); no effects of stimulation frequency, and no other interactions (*F*<1.47, NS)]. Of note, this effect was not evident in any other frequency band, including high beta, and was transient – these group differences were not present at the next time point (On25). Although this reduction in low beta power was only significant at On5, when data are pooled according to relative time point (i.e., “OFF” = BL, On30, Off60; “ON+5” = On5, On35, On55; and “ON+25” – On25, On55, On85) the effect remains (Fig. 2D) – low beta power is significantly reduced in the “ON+5” period in the HF group compared to “OFF” and “ON+25”, and compared to SHAM [(main effect of time, F_2,80_ = 5.451, p = 0.006; no effect of stimulation frequency and no interactions (*F*<1.04, NS)]. In addition, high gamma power was elevated significantly in the HF group compared to baseline at selected later time points (i.e., Off30, Off60, On85 and Off90). There were no significant within-subject differences in the SHAM group at any time point in any frequency band.

The effects on LFP activity produced by EP DBS in STR were qualitatively similar to those observed in M1 (Fig. 2A, B), with low beta power significantly reduced in the HF group at ON5 compared to BL, and compared to SHAM at On5 [significant main effect of time (F_3.54, 42.50_ = 11.97, p<0.001) and frequency band (F_1.97, 23.66_ = 5.07, p = 0.015), and a significant time × stimulation frequency interaction (F_3.54, 42.50_ = 3.54, p = 0.020); no effect of stimulation frequency and no other interactions (*F*<1.75, NS)]. As with M1, this effect was transient and frequency band-specific, and no other significant differences were present between HF and SHAM groups. Furthermore, pooled data showed similar effects as in M1; low beta power was significantly reduced in the HF group compared to SHAM at “ON+5”, and also compared to BL [main effects of time (F_2,80_ = 12.158, p<0.001) and a time×stimulation frequency interaction (F_2,80_ = 8.017, p<0.001); no effect of stimulation frequency (*F*<0.91, NS)]. However, unlike in M1, theta power in STR was significantly increased in the HF group at Off60 and Off90 compared to BL. There were no other significant within-subject effects in the HF group, and no within-subjects differences in the SHAM group.

By contrast with M1 and STR, analysis of the effects of EP DBS in LFP power in VA (Fig. 2A, B) indicated no significant differences between HF and SHAM groups in any frequency band at any time point [significant effect of time (F_2.67, 31.99_ = 13.56, p<0.001) and frequency band (F_1.10, 28.09_ = 4.43, p = 0.017); no effect of stimulation frequency and no interactions (*F*<1.86, NS)]. However, in the HF group, theta power was significantly enhanced at Off60 compared to BL. Furthermore, low beta power was significantly elevated compared to BL at Off30, On85, Off90, whereas high beta power was significantly elevated compared to BL at Off30 and Off90. There were no significant within-subject differences in the SHAM group at any time point in any frequency band.

Due to the size of EP, it was only possible to record EP LFP activity through one pole of the stimulating electrode during OFF periods. During the OFF periods, there were no significant effects on EP oscillation power in any region or band (data not shown).

Thus, HF DBS produced an early and transient reduction in low beta power compared to SHAM in M1 and STR, but not VA. Furthermore, HF DBS produced time-dependent enhancements in fast oscillation power that were region- and frequency-band specific and not present in the SHAM group.

#### Functional connectivity

**Figure 3:**
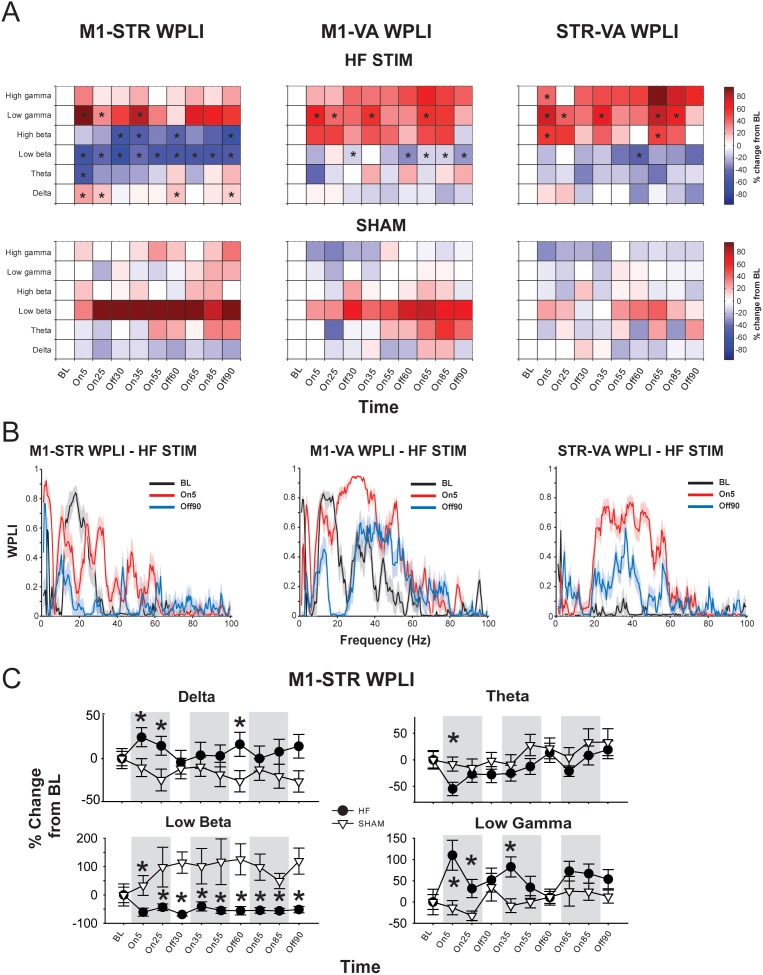
High-frequency (HF) EP DBS produces region- and frequency band-specific effects on functional connectivity. **A.** Color plots showing effects of high-frequency (top) or SHAM (bottom) EP DBS on debiased weighted phase lag index (WPLI) between regions according to frequency band and time point. * = significantly different from SHAM (p<0.05). **B.** Representative WPLI plots from a rat receiving HF stimulation at baseline (BL), in the first five minutes of stimulation (On5), and after 90 minutes of stimulation (Off90). Shaded area represents 95% confidence interval generated using jackknife statistics. **C.** Effects of HF and SHAM EP DBS on M1-STR WPLI over time. Error bars represent S.E.M. * = significantly different from SHAM (p<0.05).

EP DBS also produced widespread changes in functional connectivity along the circuit, as assessed using WPLI (Fig. 3). M1-STR WPLI was significantly increased in the HF group compared to SHAM in the delta (at On5, On25, and Off60) and low gamma bands (at On5, On25, and On35); and significantly decreased in the theta (at ON5), low beta (all time points) and high beta (at Off30, On35, Off60, and Off90) [significant frequency band × stimulation frequency interaction (F_2.84, 34.08_ = 7.803, p = 0.001); no other main effects or interactions (*F*<2.09, NS)]. There were no within-subject effects in either group.

EP DBS also produced effects on M1-VA connectivity (Fig. 3A, B). Low beta band WPLI was significantly decreased in the HF group compared to SHAM at Off30, Off60, On65, On85, and Off90 [significant frequency band × stimulation frequency interaction (F_2.53, 30.38_ = 2.75, p = 0.042); no other main effects or interactions (*F*<1.96, NS)]. In addition, WPLI in the low gamma band was significantly higher in the HF group than the SHAM group at On5, On25, On35, and On65. In SHAM animals, there was a general enhancement of synchronization in the low beta band relative to baseline and other frequency bands, but this effect did not reach significance; there were no within-subject effects in either group.

Analysis of the effects of EP DBS on STR-VA connectivity (Fig. 3A, B) indicated that HF DBS produced significant increases in WPLI in the high beta (at On5 and On65), low gamma (at On5, On25, On35, On65, and Off85) and high gamma (at On5, On85) bands, and a significant decrease in low beta synchronization at Off60. [significant frequency band × stimulation frequency × time interaction (F_5.64, 67.69_ = 2.46, p = 0.025); no effects of time or frequency band, and no other interactions (*F*<1.30, NS)]. There were no within-subject effects in either group.

As mentioned above, it was only possible to record EP LFP activity (and WPLI between EP and the other regions) during OFF periods. In the HF group, EP-M1 WPLI was significantly reduced compared to SHAM in the delta band (at Off30, Off60, and Off90) as well as the low beta band at Off60 and Off90 [significant main effect of stimulation frequency (F_1.22, 14.65_ = 4.386, p = 0.048)], and a significant time × frequency band interaction (F_3.89, 46.73_ = 2.101, p = 0.025); no effect of time, frequency band, and no other interactions (*F*<1.32, NS)]. There were no within-subject effects in either group.

EP-STR coherence was also affected by EP DBS [significant band × stimulation frequency (F_1.41, 16.96_ = 1.922, p = 0.039) interaction, no other significant main effect or other interactions (*F*<1.29, NS)]. Post-hoc analysis showed a significant reduction in WPLI in the HF group compared to SHAM in the theta (at Off30) and low beta (at Off60) bands. There were no within-subject effects in either group.

With respect to EP-VA connectivity, there was a significant stimulation frequency × time × frequency band interaction (F_5.81, 46.73_ = 2.170, p = 0.029); WPLI was significantly reduced in the HF group compared to SHAM in both the delta and low beta bands (at Off 30 and Off 90).

Thus, the predominant effect of HF EP-DBS on functional connectivity was desynchronization in the low beta band, initially only between M1 and STR, but between all other regions with sustained stimulation, and enhanced synchronization in the low gamma band that was most prominent between VA and other regions along the circuit. Interestingly, enhanced gamma connectivity was only observed when stimulation was on; beta desynchronization persisted into OFF periods.

### Stimulus-induced Oscillations

**Figure 4:**
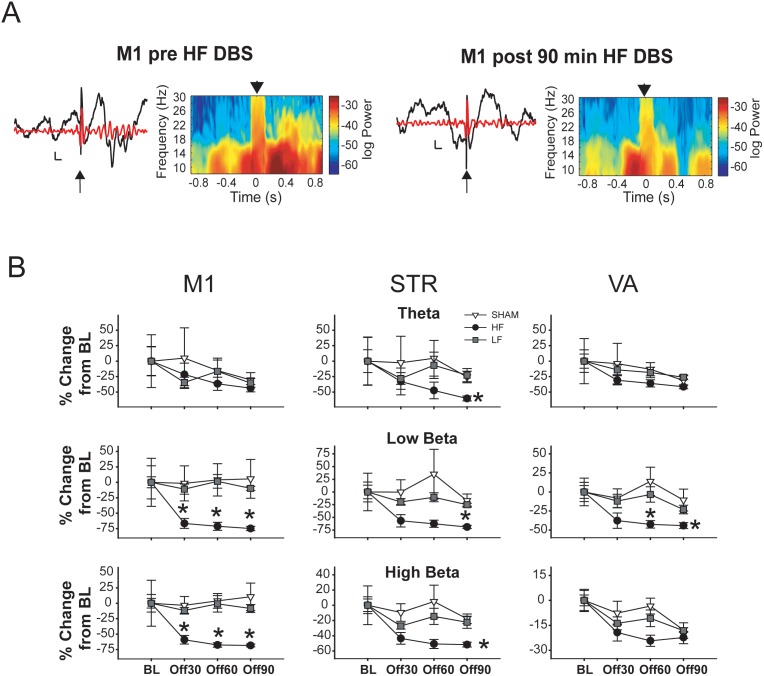
High-frequency (HF) EP DBS suppresses acute EP-induced beta oscillations in all recorded regions. **A.** Representative raw (black) and filtered (6–30 Hz; red) voltage traces from M1 (calibration: 200 ms, 0.2 mV) and time-frequency spectrograms showing effects of acute EP stimulation before (left) and after (right) 90 minutes of high-frequency (HF) EP DBS. **B.** Changes in induced oscillation power according to region and time point. Low-frequency (LF) EP DBS does not suppressed induced oscillations as HF DBS does. Error bars represent S.E.M. * = significantly different from SHAM and LF (p<0.05).

Acute EP stimulation often resulted in induced oscillations (i.e., not time-locked to the stimulus; see Fig. 4A) at all recording sites. Changes in induced oscillations as a function of time and stimulation frequency were quantified by examining the spectral power within regions and coherence between regions in the 1 s following stimulation, normalized to activity in the 1 s prior to stimulation during the four OFF periods.

#### Induced Power

In M1, EP DBS had a number of effects on induced LFP power (Fig. 4). Induced low and high beta power were both significantly reduced in the HF group compared to both SHAM and LF groups at all three OFF time points (Off30, Off60, Off90) [significant main effects of stimulation frequency (F_2, 19_ = 3.633, p = 0.046), time (F_1.96, 37.28_ = 3.559, p = 0.039), and frequency band (F_2.09, 39.76_ = 3.466, p = 0.039), as well as a significant stimulation frequency × frequency band interaction (F_4.19, 39.76_ = 8.040, p<0.001); but no other interactions (*F*<2.01, NS)]. There were no significant between-group differences in any other frequency bands at any other time points. In addition, induced power in the low and high beta bands was significantly reduced in the HF group at all time points compared to BL. No significant within-subject effects were observed in the other groups at any time point.

The effects of EP DBS on induced oscillatory activity in STR were largely similar to those observed in M1 (Fig. 4). Induced theta power was significantly reduced in the HF group compared to both SHAM and LF at Off90, whereas induced low and high beta were both significantly reduced compared to SHAM (but not LF) at Off30 and Off60, and significantly reduced compared to both LF and SHAM at Off90 [significant main effect of time (F_2.08, 39.48_ = 6.44, p = 0.003), and a significant stimulation intensity × frequency band interaction (F_4.47, 42.43_ = 4.196, p = 0.005); no effect of stimulation frequency or frequency band, and no other interactions (*F*<2.39, NS)]. In the HF group, compared to BL there was significant reduction in low beta power at Off30 and Off90, a significant reduction in high beta power at all 3 time points, and a significant reduction in low gamma power at Off90. No within subject effects were observed in the LF and SHAM groups.

Analysis of the effects of EP DBS on induced LFP activity in VA (Fig. 4) revealed that induced low beta power was significantly attenuated compared to LF and SHAM at OFF60 and OFF90 [significant main effects of time (F_3, 57_ = 7.651, p<0.001), and frequency band (F_2.23, 42.28_ = 3.855, p = 0.025); no effect of stimulation frequency and no interactions (*F*<2.23, NS)]. Furthermore, induced high beta power was reduced compared to SHAM but not LF at Off60. With respect to within-subject effects, in the HF group there was a significant decrease in both induced low and high beta at all time points compared to BL. In the LF group, there was a significant reduction in induced high beta at Off90 compared to BL, and in induced high gamma at Off30 compared to BL. There were no significant within-subject effects in the SHAM group.

Thus, compared to SHAM and LF, HF EP DBS produced a robust decrease in evoked oscillatory activity that was predominantly confined to the beta band (both low and high). While small differences were apparent, these effects were for the most part similar in all recorded areas. LF stimulation produced effects that were either similar in direction as the changes produced by HF stimulation (i.e., in VA) or not different from SHAM or LF BL (i.e, in M1 and STR).

## Discussion

EP DBS delivered for 90 minutes generated widespread alterations in spontaneous and stimulus-induced LFP oscillations along a motor cortical-basal ganglia-thalamic circuit known to exhibit pathological activity in PD and dystonia (summarized in Table 1). The most prominent effect was a reduction in beta synchronization and enhancement of gamma synchronization; however, although some effects appeared similar across regions, others occurred in a region- and frequency band-specific manner, and importantly, many evolved over time. We also observed a dissociation of the effects of DBS on power within a region and functional connectivity between regions. Although EP DBS produced an initial transient reduction in power in the low beta band in M1 and STR, synchronization between these two regions in this band was dramatically reduced at all time points, and EP DBS markedly suppressed the ability of acute EP stimulation to induce beta oscillations in all regions along the circuit. These stimulus-induced effects were specific to HF DBS and were not present with LF (i.e. non-therapeutic) DBS. Gamma synchronization between regions along the circuit was enhanced when DBS was on, and with time, sustained DBS enhanced power in the faster frequency bands.

**Table 1:** Summary of major effects.

	M1			STR			VA		
	Spont	Evoked		Spont	Evoked		Spont	Evoked	
POWER	HF	HF	LF	HF	HF	LF	HF	HF	LF
Slow/delta	–	–	–	–	–	–	–	–	–
Theta	–	–	–	↑	–	–	↑	–	–
Beta	**↓**	**↓**↓	–	**↓**	↓	–	↑	↓	–
Gamma	↑	–	–	–	–	–	–	–	–
	**M1-VA**			**M1-STR**			**STR-VA**		
**WPLI**	**Spont HF**			**Spont HF**			**Spont HF**		
Slow/delta	**↑**↑			–			–		
Theta	**↓**			–			–		
Beta	**↓**↓			↓			↓		
Gamma	**↑**			**↑**↑			**↑**↑		

Arrows signify significant increases or decreases (p<0.05) in oscillation power or functional connectivity (assessed using debiased weighted phase lag index; WPLI). Bold arrows represent early effects and thin arrows represent late effects, which are more widespread.

### Neural circuitry

The aim of this study was to assess the effects of EP DBS simultaneously in a number of regions that comprise a primary motor circuit. The direct efferent pathway from the striatum projects directly to the entopeduncular nucleus [53], [54], which in turn projects to the VA/VL thalamus [55], [56]. Thalamocortical projections to primary motor cortex have been well-studied (Jones, 2005) as have projections from M1 to the striatum [57], [58]. Together, these regions are proposed to create a functional segregated motor processing loop [59]. In rats and pigs, respectively, EP DBS produced changes in immediate early gene expression [60] and BOLD imaging [61] in all regions along this circuit; GPi DBS in primates has been shown to produce effects consistent with activation of GPi axons [19], [20], and modulates neuronal firing in M1 [62]. The present work allowed us to examine how circuit-wide changes in neural activity evolve over time and allowed comparison to human DBS studies that report changes in LFP oscillations.

### LFP oscillations, movement disorders and DBS

Accumulating evidence suggests that DBS may exert beneficial effects by reducing pathological beta synchronization. Thus, prominent oscillatory activity in the beta band throughout the basal ganglia and motor cortex is reported in PD patients off dopaminergic medications [63]–[66], as well as animal models of PD that involve destruction of dopamine neurons [67]–[70]. Moreover, L-DOPA administration reduces beta oscillation power concomitantly with movement facilitation [46], [63], [71], and beta desynchronization is associated with movement initiation [72]–[74]. While beta oscillations are associated with akinesia/bradykinesia, gamma oscillations are generally associated with movement, with movement-related gamma synchronization found in a number of regions [75]–[77]. Furthermore, in PD patients the prokinetic effects of levopoda are accompanied by increases in gamma power [63], [64], [78], [79].

The relationship between synchronization in BG and dystonia is less clear. LFPs recorded from the BG of dystonia patients display oscillation peaks at a lower frequency than PD patients, typically at 4–10 Hz [80]–[84]. In dystonia there is also less coherence between LFP oscillation and single neuron firing than in PD patients [85]. These oscillations are coherent with [86] and thought to drive [87] EMG oscillation in the affected muscle groups; using a sensory trick to alleviate dystonic contractions is associated with desynchronization in this frequency band [83].

DBS of the subthalamic nucleus (STN) has been shown to reduce BG beta oscillations in PD patients either during stimulation or in the period immediately following stimulation, while therapeutic effects persist [88]–[94]. Although the effects of GPi DBS on oscillatory activity are not well studied, GPi DBS suppresses oscillatory activity in the beta range in MPTP-treated macaques [95].

### EP DBS and LFP oscillations

Based on this literature, we hypothesized that EP DBS would promote desynchronization, particularly in the lower frequency bands. While this was indeed the case, the effects we observed were more complex, including enhancements in gamma synchronization, and dissociations between changes in power within a region and synchronization between regions. While DBS produced an initial reduction in low beta power in both M1 and STR, this effect was transient and did not persist over sustained stimulation. However, synchronization in this band between these two regions was profoundly suppressed at all time points. DBS also enhanced low gamma synchronization between regions throughout the circuit, particularly between VA and other regions, and with longer stimulation, DBS enhanced oscillation power in the faster frequency bands within both M1 and VA. The SHAM group displayed a gradual non-significant enhanced broadband power over time. This effect may be due to changes in network activity stemming tissue damage during electrode insertion, or is a side effect of urethane anesthesia. We believe the latter is more probable, as urethane has a variety of effects on neural activity that could contribute to the observed power changes, including effects on catecholamine levels and ion channel function [96], [97] (Maggi and Mello, 1986; Hara and Harris, 2002). The reduction in low beta power and functional connectivity that we observed is consistent with the ability of therapeutic DBS to reduce pathological beta oscillations in PD patients. The fact that significant changes occurred in the low beta band only is also consistent with specific reduction in this band following L-DOPA administration and associated with movement facilitation [46]–[48]. Spontaneous measures represent the general activity state of the system; stimulated responses provide an index of how the system responds to activation. We saw a pronounced effect of HF DBS but not LF DBS on the ability of acute stimulation of the EP to induce beta oscillation in all recorded regions. This effect is consistent with the desynchronization of spontaneous beta oscillations and connectivity seen with DBS.

LF stimulation of the STN is reported to worsen movement performance on a variety of tasks [33]–[35]. We expected LF EP DBS to produce effects opposite in direction to those produced by HF EP DBS, however, LF stimulation either produced no significant effects, or in the same direction as HF DBS but reduced in magnitude. This may have occurred because we applied the same amplitude of DBS for both LF and HF, resulting in overall lower current injection into the tissue with LF [98].

### Time-dependent effects of EP DBS

Our most notable finding is the differential time course of the observed changes in LFP activity (i.e., early vs. late). To our knowledge, virtually all studies examining the effects of DBS on neuronal firing or LFP oscillations have focused on the short-term effects of stimulation, on a time scale ranging from seconds to a few minutes. While understanding the initial effects of stimulation is essential, DBS is applied chronically over years, and the effects of DBS may evolve over time. For example, in PD patients, although therapeutic effects are seen within minutes of DBS application [27], not all symptoms respond equally quickly to DBS [1] and DBS for dystonia can take months to years to achieve maximum benefit [28]–[30]. The early low beta desynchronization in (and between) M1 and STR we report here ma­y be similar to changes seen immediately in PD patients and together with enhanced gamma connectivity may be sufficient for facilitating voluntary movement. The more slowly developing beta desynchronization between the other regions and enhancement of faster oscillation power along the circuit may represent the early stages of the plastic reorganization that relate to the therapeutic effects of GPi DBS in dystonia [32]. The enhanced fast oscillatory synchronization we observed throughout this motor circuit may act as a stabilizing influence, promoting coordinated physiological information flow throughout the circuit, and dampening the effects of pathological synchronization in lower frequency bands.

There are several limitations to this study. One is that our “extended” stimulation remains considerably less than days or weeks; however, the time-dependent changes observed here may represent the initial stages of a plastic or compensatory response to the hyperacute effects of DBS. Two other limitations are the use anesthesia and normal intact animals. Even with anesthetics like urethane that are thought to preserve many aspects physiological responses, neuronal activity and the synchronization thereof are likely to be differently regulated in the awake and anesthetized states (Hara and Harris, 2002). Several studies support the relevance of our data. Dopamine-depleted rats show prominent spontaneous cortical and subcortical beta oscillations in both awake and anesthetized states (Mallet et al., 2008a, Mallet et al., 2008b). Furthermore, Ahrens and Freeman (2001) reported similar profiles of electrically-evoked entorhinal cortex LFP activity in awake and anesthetized rats. In normal animals it can be different to ensure the stimulation applied is in the appropriate therapeutic range. Preliminary work showed that the stimulation parameters used caused no motor side effects in normal animals, produce a charge density approximating what is clinically effective in humans (Fakhar et al., 2013), and produced effects in various rat behavioral studies (Creed et al., 2011, Creed et al., 2012a), suggesting our parameters are consistent with those used in the preclinical and clinical literature. The question remains as to whether the effects on synchronization that we observe in normal animals are relevant and generalizable to disease states. Given that the effects of EP/GPi DBS on oscillatory activity have not been characterized in intact animals, and that GPi DBS is clinically effective for both dystonia and PD, diseases with unique pathological profiles, the findings reported here will help identify how responses to DBS may vary under different physiological and pathological conditions. Furthermore, the reduction and enhancement beta and gamma synchronization, respectively, is consistent with what is observed in the clinic with DBS for PD (Wingeier et al., 2006, Kuhn et al., 2008, Bronte-Stewart et al., 2009, Eusebio et al., 2011, Giannicola et al., 2012).

### Implications

The effects of DBS on LFP activity are not static, but rather reflect an evolving pattern of effects that vary by time, frequency band, and region. In addition, the widespread and specific alterations in oscillatory activity provide further evidence that DBS can modulate neural activity distant to the stimulated nucleus; indeed, our results point to a constellation of changes across the entire cortico-BG-thalamic circuit. The time-dependent effects DBS are likely to be most relevant for conditions that show a more gradual response to stimulation, and suggest that studies of therapeutic mechanisms should examine the effects of both short-duration and more extended stimulation.

## Supporting Information

Figure S1 **Representative voltage traces and power spectrum during HF (left) and LF (right) DBS, before (top) and after (bottom) stimulus artifact removal.** Note the acute responses to stimulation in the LF trace that contaminate the power spectrum even after artifact removal.
(EPS)
